# Complete Genome Sequences of *Edwardsiella tarda*-Lytic Bacteriophages KF-1 and IW-1

**DOI:** 10.1128/genomeA.00089-12

**Published:** 2013-02-07

**Authors:** Motoshige Yasuike, Emi Sugaya, Yoji Nakamura, Yuya Shigenobu, Yasuhiko Kawato, Wataru Kai, Atushi Fujiwara, Motohiko Sano, Takanori Kobayashi, Toshihiro Nakai

**Affiliations:** a Research Center for Aquatic Genomics, National Research Institute of Fisheries Science, Fisheries Research Agency, Yokohama, Japan; b Department of Marine Biotechnology, Faculty of Life Science and Biotechnology, Fukuyama University, Fukuyama, Hirosima, Japan; c Research Center for Fisheries Oceanography and Marine Ecosystem, National Research Institute of Fisheries Science, Fisheries Research Agency, Yokohama, Japan; d Graduate School of Biosphere Science, Hiroshima University, Higashi-Hiroshima, Japan

## Abstract

We report the complete genome sequences of two *Edwardsiella tarda*-lytic bacteriophages isolated from flounder kidney (KF-1) and seawater (IW-1). These newly sequenced phage genomes provide a novel resource for future studies on phage-host interaction mechanisms and various applications of the phages for control of edwardsiellosis in aquaculture.

## GENOME ANNOUNCEMENT

*Edwardsiella tarda*, a Gram-negative bacterium, has been isolated from a wide variety of animals, including humans. Particularly, *E. tarda* has long been known as a pathogen of various diseases causing severe economic losses in cultured fish species such as channel catfish (*Ictalurus punctatus*), Japanese eel (*Anguilla japonica*), and Japanese flounder (*Paralichthys olivaceus*) (reviewed in reference 1). No antimicrobial agent against *E. tarda* infections of fish is currently licensed in Japan. Although various types of experimental *E. tarda* vaccines have been reported, the resistance of *E. tarda* to phagocyte-mediated killing (2) has made it difficult to develop an effective vaccine (reviewed in references 1 and 3).

A number of bacteriophages (phages) that infect *E. tarda* have been isolated from flounder tissues and seawater samples from fish farms (4). The potential use of *E. tarda* phages as therapeutic agents against edwardsiellosis and as indicators of the presence of *E. tarda* in culture environments has been suggested (5). Genomic information regarding *E. tarda* phages is important for understanding phage-host interactions as well as for applications of the phages for the control of disease. In the present study, we determined the complete genome sequences of phages isolated from Japanese flounder kidney (KF-1) and seawater (IW-1).

Electron microscopic observations of both KF-1 and IW-1 indicated that each phage was a member of the family *Podoviridae* (6). Whole-genome shotgun sequencing of KF-1 and IW-1 was performed by using Roche 454 GS-FLX titanium pyrosequencing. *De novo* assembly of sequence reads was performed by using a 454 Newbler 2.5.3, and open reading frames (ORFs) were predicted by using GeneMarkS (7) and Glimmer3 (8). The predicted ORFs were annotated by using BLASTP (9) against the viral sequence database (E value threshold of 1E^-3^) and InterProScan (10). The potential presence of tRNAs was scanned by using the tRNAscan-SE 1.21 (11).

The sizes of the two entire genomes were 41,549 bp for KF-1 and 41,684 bp for IW-1. These sequences showed approximately 99% identity at the nucleotide level, with a GC content of 48.3%. Forty-eight ORFs were predicted for both genomes. Of these 48 ORFs, only 12 ORFs (25.0%) matched with at least one entry in the viral sequence database. Furthermore, a high level of sequence divergence was observed between these 12 annotated ORFs and the other phage proteins (range from 26 to 55% identities). A preliminary maximum-parsimony phylogenetic analysis based on large terminase subunit genes showed that KF-1 and IW-1 are closely related to the family *Podoviridae*. Thus, this preliminary phylogenetic analysis coincides with our morphological observations by electron microscopy. These newly sequenced *E. tarda* phage genomes and existing *E. tarda* genome sequences (12, 13) will provide the genetic resources for future studies on phages and edwardsiellosis.

### Nucleotide sequence accession numbers.

The complete genome sequences of the two *E. tarda* phage isolates were submitted to DDBJ under accession numbers AB757800 (KF-1) and AB757801 (IW-1).
